# The Dilution Effect and Information Integration in Perceptual Decision Making

**DOI:** 10.1371/journal.pone.0138481

**Published:** 2015-09-25

**Authors:** Jared M. Hotaling, Andrew L. Cohen, Richard M. Shiffrin, Jerome R. Busemeyer

**Affiliations:** 1 Department of Psychology, University of Basel, Basel, Switzerland; 2 Department of Psychology, University of Massachusetts, Amherst, Massachusetts, United States of America; 3 Department of Psychological and Brain Sciences, Indiana University, Bloomington, Indiana, United States of America; University of Melbourne, AUSTRALIA

## Abstract

In cognitive science there is a seeming paradox: On the one hand, studies of human judgment and decision making have repeatedly shown that people systematically violate optimal behavior when integrating information from multiple sources. On the other hand, optimal models, often Bayesian, have been successful at accounting for information integration in fields such as categorization, memory, and perception. This apparent conflict could be due, in part, to different materials and designs that lead to differences in the nature of processing. Stimuli that require controlled integration of information, such as the quantitative or linguistic information (commonly found in judgment studies), may lead to suboptimal performance. In contrast, perceptual stimuli may lend themselves to automatic processing, resulting in integration that is closer to optimal. We tested this hypothesis with an experiment in which participants categorized faces based on resemblance to a family patriarch. The amount of evidence contained in the top and bottom halves of each test face was independently manipulated. These data allow us to investigate a canonical example of sub-optimal information integration from the judgment and decision making literature, the *dilution effect*. Splitting the top and bottom halves of a face, a manipulation meant to encourage controlled integration of information, produced farther from optimal behavior and larger dilution effects. The Multi-component Information Accumulation model, a hybrid optimal/averaging model of information integration, successfully accounts for key accuracy, response time, and dilution effects.

## Introduction

Information integration refers to the combination of different sources of information for the purpose of performing some task. To comprehend speech, for instance, one can attend to both its visual and auditory components. As another example, to produce an accurate diagnosis, a doctor needs to consider the patient’s symptoms, family history, diet, and exercise habits. Each source of information, on its own, provides some predictive or diagnostic value, but integrating these sources will usually yield better performance. The manner in which these sources are integrated will determine the probability of success.

The extant literature provides numerous examples in which observers combine multiple sources of information in a near-optimal fashion. There are just as many examples, however, in which integration is far from optimal and clearly follows simple heuristics. The stimuli and tasks for which these divergent results are found often differ qualitatively. Tasks involving perceptual processing, for example, speech comprehension [1], seem to be less susceptible to the use of heuristics than tasks involving more quantitative stimuli, for example, probability judgments [2]. The present research directly tests this claim and explores the modulating effect of stimulus presentation on the use of heuristics in a uniform information integration paradigm. In particular, the current research tests the hypothesis that sources of information that are easier to perceptually combine will be more resistant to the dilution effect, a robust information integration phenomenon whereby additional diagnostic information actually decreases accuracy.

### Information Integration in Judgment and Decision Making

There is abundant evidence from many studies of judgment and decision making that information from different sources is often integrated via heuristic strategies. Sometimes simple heuristics produce results that approach optimal decision making [3], but in many other cases the results fall well short of optimal combination, relative to standard rational theories of inference [4]. Findings of deviations from rational behavior have had a great influence on theorizing, and for a large subset of judgment researchers it is now common practice to assume sub-optimal performance as a starting point for theories of information integration.

The conjunctive fallacy, unpacking effect, and reliance on rule-based heuristic strategies are a few of the many common findings in the judgment literature that violate normative models of information integration. The conjunctive fallacy [5, 6] is typically illustrated with the famous “Linda the bank teller” story. The laws of probability imply that the probability of a conjunction of two events cannot be larger than the probability of either event separately: e.g., P(X∩Y | Z) ≤ P(X | Z). Yet a majority of respondents across a variety of studies claimed that statements such as “Linda is a bank teller and is active in the feminist movement” to be more likely than “Linda is a bank teller.” The unpacking effect relates to the finding that breaking an event into its component parts makes the event seem more likely [7]. According to common normative models, there should be no such increase in likelihood. Finally, there are many examples of reliance on rule-based heuristic strategies in tasks of judgment. One of the most compelling is the “Take The Best” heuristic [8]. According to this decision making algorithm, the dimensions along which a number of choices vary are ranked according to their predictive validity. Rather than incorporating all cues into a judgment according to their relative validity, a selection is made based simply on the values of the most valid discriminating dimension. In these situations (and many more) observers have been shown to violate rational principles of information integration.

### Information Integration in Perception, Categorization, and Memory

In contrast to the judgment literature, there have been numerous highly successful applications of optimal or rational models of information integration in fields such as perceptual categorization and perception. In this article, we use the word *optimal* in a Bayesian statistical sense (note that some have argued that Bayesian methods do not always guarantee optimal performance [9]). The success of such models has moved researchers in these more perceptual fields to begin with an assumption of optimality and only later investigate sub-optimal or heuristic-based performance. Examples of optimal information integration are easy to find in these more perceptual domains, and the following are a very few of many potential examples. Ashby and colleagues [10–12] have found categorization performance to be well described by a decision bound that either nearly optimally integrates information across two dimensions or uses a rule of the same form as the optimal bound. Using an exemplar model of categorization, Nosofsky [13] suggested that observers tend to distribute attention among dimensions so as to optimize categorization performance. Tenenbaum [14, 15] has successfully utilized a Bayesian framework for modeling human concept learning. Anderson [16] has demonstrated that many common results in both the categorization and memory literatures can be well described by his Bayesian rational model. The Fuzzy Logic Model of Perception [1, 17] is based on the idea that observers optimally integrate different sources of information and has been applied successfully to a large range of data.

In sensory science, optimal information integration is usually the default assumption and the starting point for research. Of thousands of potential examples, we mention Burgess, Wagner, Jennings, and Barlow [18] who found the ability of human observers to discriminate visual patterns in noisy backgrounds to be very close to that of an ideal observer. Geisler and Diehl [19] used a Bayesian framework to analyze the relationship between statistical properties of the environment and the evolution of the cognitive and perceptual systems. Kersten, Mamassian, and Yuille [20] provide a review of recent developments in using Bayesian approaches to model people’s ability to perceive objects in complex and noisy environments.

### The Dilution Effect

Although information integration is an object of study by researchers in both judgment and decision making domains and in cognitive and sensory domains, these fields often seem to be operating independently of each other. One source of such independence is the wide difference in experimental paradigms. The judgment literature focuses mainly on linguistic or quantitative statements of probabilities, and is concerned with the ways in which a person uses information to assess, estimate, and infer what events will occur [21]. The other lines of research discussed above typically rely on more perceptual stimuli, such as images and sounds, and concentrate on how the information is produced from external stimulation, often with reference to particular episodes.

This article focuses on one phenomenon in which sub-optimal information integration has often been observed: *the dilution effect*. There are several forms of this effect, but the general finding is that adding null or weak positive evidence to what is already very strong positive evidence reduces the overall strength of belief about a hypothesis. This “dilution” of the strong evidence with weaker evidence suggests the use of a simple averaging heuristic. The dilution effect has been replicated in numerous judgment and decision making studies [22, 23] and in such diverse areas as legal [24] and social [25] reasoning.

Shanteau [26] gives one of the earliest demonstrations of the dilution effect. In this study, the experimenter drew samples of red (R) and white (W) beads with replacement from one of two boxes. The 70/30 box had 70% white beads and 30% red beads. The 30/70 box had 30% white beads and 70% red beads. The participants did not know from which box the beads were drawn. In one condition, the experimenter drew the sequence WWWRWR from one of the boxes. After every two beads, the participants were asked to estimate the probability that the beads came from the 70/30 box. The mean judgments after WW, WWWR, and WWWRWR were 69.3%, 64.0, and 60.6, respectively. The WW sample provides diagnostic information that clearly points to the 70/30 box. However the subsequent samples are nondiagnostic; they could have come from either box with equal probability, and should not have changed the estimated likelihood that the entire sequence came from the 70/30 box. Yet this non-diagnostic information caused the estimated probability to drop.

A variation of an example from McKenzie, Lee, and Chen [24] presents a different and naturalistic illustration of the dilution effect. Imagine a defendant standing trial on criminal charges: John Smith is accused of robbing a bank. The prosecution first calls a witness that presents a strong case for the defendant’s guilt: she is confident that she saw John Smith rob the bank. The prosecution then calls a second witness who did not get a clear view and claims only to have seen a male of John Smith’s height and race rob the bank. Although this evidence is weaker, it should increase rather than decrease belief in the defendant’s guilt because the description fits John Smith and tends to rule out many other potential robbers. Judgments of guilt, however, usually decrease after hearing this weakly positive evidence.

### Perceptual Stimuli

The current research explores the dilution effect using perceptual stimuli, which impart a number of advantages (also see [27]). First, issues of interpretation and language understanding do not come into play. Numerous papers have suggested that participants often do not properly understand what is asked of them when presented with standard judgment and decision making stimuli. For example, the conjunction rule is violated less often if participants interpret “Linda is a bank teller” to mean that she is a bank teller and *not* active in the feminist movement (see [5] for a short review). Furthermore, observers often misinterpret probability values, and sometimes perform more effectively when data are, instead, presented as frequencies [28]. In contrast, the present task simply requires perceptual matching, thereby greatly reducing any confounding influence of language conventions or mathematical training.

Although the dilution effect has commonly been studied using traditional judgment and decision making stimuli, it easily lends itself to exploration in a perceptual setting. The present research explores the combination of weak and strong evidence from different parts of a face. To continue the above example, say you are trying to identify whether a face captured on a security camera is the defendant John Smith. The photo is indeed that of John Smith, but only the top half of the face is relatively clear; the bottom half is in partial shadow and harder to see. The top and bottom halves of the face lend strong and weak evidence to the decision, respectively. In this way, using face stimuli allows for precise control of the amount of information in an image.

To preview the current experiment, the appearance of each test face stimulus is manipulated by morphing two target faces representing patriarchs of the Jones and Smith families. A morph comprised of 90% Jones and 10% Smith would provide very strong evidence that the test face belonged to the Jones family. Likewise, a 60% Jones and 40% Smith morph would provide only weak support for Jones. The observer’s goal is to categorize the faces into one of the two families, based on resemblance. Critically, the top and bottom halves of the test face are morphed independently and so provide different levels of support for the two families. The key idea is that, in direct analogy to standard work on the dilution effect, the two half faces act as two sources of information to be combined before making a choice.

The primary goal of this research is to determine whether the two sources of information in a face combine in a near-optimal fashion, as predicted by models of perceptual integration, or whether the information will be combined in a sub-optimal manner, as exemplified by the dilution effect. Using perceptual stimuli allows us to bridge the methodological divide between these areas through use of a common experimental paradigm and uniform methods of analysis.

### Automatic and Controlled Processing

How will observers combine the information contained in each half face? Analogous to the conflicting results from perceptual and judgment and decision making paradigms, the answer may depend, in part, on the type of processes that are engaged. As a first step towards addressing this issue, we hypothesize that information integration is more susceptible to the dilution effect when information is combined by controlled, cognitive processes than when it is combined by automatic, perceptual processes.

Distinguishing automatic from controlled processing is very common, both in theory and in empirical research. Whereas automatic processing is usually assumed to be fast and independent of conscious manipulation, controlled processing is assumed to be slow and conscious. Although less flexible, automatic processing is usually assumed to be more robust, less prone to large errors, less based on heuristics, and closer to optimal than controlled processing (although, see for example [3, 29]).

Theories differentiating automatic and controlled processing are common in both judgment and decision making (e.g., [21, 30–35]) and in perceptual categorization (e.g. [36–38]), memory (e.g. [39]), and perception (e.g. [40–44]).

Although both automatic and controlled processes are typically engaged in most judgment and decision making and perceptual tasks, the contrasting information integration paradigms discussed previously can be seen as prototypes for tasks encouraging automatic and controlled processes. That is, whereas efficiently matching visual stimuli, faces in particular, is a highly automated task [45, 46], the integration of quantitative values is often deliberative and controlled [8, 47, 48]. This distinction suggests that the stimuli used in traditional dilution effect paradigms may be more likely to encourage heuristic processing. Closer to optimal integration is more likely for tasks that invite automatic processing.

Another benefit of using perceptual stimuli is that the test faces can be designed to bias processing toward controlled or automatic processing in a common experimental paradigm with minimal variation across conditions. In one set of conditions, the *together* conditions, the two half faces are shown atop one another, in a normal configuration. Because identification of faces is an over-learned task, these conditions should promote automatic processing and were predicted to be less prone to simple heuristics. That is, in contrast to the dilution effect, weak evidence in the together conditions, when added to strong evidence from the same category, should increase classification accuracy. In a second set of conditions, the *split* conditions, the two half faces were separated horizontally. Because our perceptual systems have rarely dealt with the need to combine face parts separated horizontally in space, it seems likely that each half face would be processed separately, and that the results would later be combined with controlled strategies. If so, the well-known cognitive difficulties of combining information might come into play, encouraging heuristic use. That is, weak and strong evidence should produce a dilution effect.

### A Perceptual Information Integration Experiment

The goal of this experiment is to demonstrate dilution effects using perceptual stimuli (i.e., faces) and to determine whether dilution is modulated by stimulus presentation format. In particular, this experiment tests whether two sources of information that are combined in a more controlled, rather than automatic, manner will be more likely to produce dilution.

On each trial, two target faces representing the Jones and Smith family patriarchs were displayed on either side of a test face created by morphing the targets. Depending on the proportion of the Jones and Smith target present in the morph, the top and bottom halves of the test faces provide weak, medium, or strong evidence for either the Jones or Smith family. Test faces were either a half face (top or bottom) or a whole face (both top and bottom). The whole faces were presented either split to promote controlled processing (top and bottom half faces shifted horizontally) or together to promote automatic processing (top and bottom half faces in a normal configuration). The observer’s task was to determine which target better matched the test face.

Based on the many studies showing near-optimal combination of perceptual information, in the together condition, two half faces favoring Jones should produce even stronger Jones responses relative to either half face alone. Alternatively, as in more traditional judgment and decision making studies, in the split condition, weak evidence might detract from strong evidence to produce a dilution effect. That is, splitting a face, which disrupts normal face processing, should result in greater dilution than when the face is presented normally.

As discussed previously, the dilution effect is typically defined as non-optimal information integration in which weak evidence reduces the influence of strong evidence. It is important to note, however, that this definition is a strong form of dilution, originating from the standard dilution effect paradigm in which strong evidence is combined with weak or neutral evidence (e.g. [23]). The current work extends this paradigm by allowing for a weaker form of dilution. In particular, weak evidence may not reduce the influence of strong evidence, but should still combine less efficiently for the split faces relative to the together faces. The present study also generalizes the standard paradigm by factorially combining weak, medium, and strong evidence.

In addition to empirically testing for the presence of dilution, we also evaluate a model of information integration that might explain such data. We propose the *Multi-component Information Accumulation* model to account for our findings. This model describes a process by which information is repeatedly drawn from multiple sources during deliberation until a decision threshold is reached. The model simultaneously accounts for choice proportions and response times, providing insight into the possible cognitive processes that might underlie information integration in our study.

## Method

### Ethics Statement

This study, and its procedure for obtaining consent, was approved by the Institutional Review Board at the Office of Research Administration at Indiana University Bloomington (protocol 07–12475). All participants read and signed an informed consent form before beginning the experiment.

### Participants

Nineteen students from Indiana University (undergraduate and graduate) were paid $10 per hour to participate in this study. Participants received points based on performance. The participant with the highest performance received a $20 bonus.

### Stimuli

First, two target faces were generated. Second, these target faces were used to calibrate the weak, medium, and strong half faces individually for each participant. Finally, these half faces were used to create the test faces.

Morphing will be used to create the weak, medium, and strong half faces. In this context, a morph is a linear combination of the gray-scale values at every pixel from two images. For example, consider a morph between the Jones and Smith faces. Each pixel of the 75% Jones morph is determined by weighting the gray-scale value of each pixel in the Jones and Smith faces by .75 and .25, respectively, and then adding the values.

The following notation is used to describe the stimuli. Weak, medium, and strong half faces are denoted as w, m, and s, respectively. A weak, medium, and strong top half face is designated as w/, m/, and s/, respectively. Likewise, bottom half faces are designated as /w, /m, and /s. Whole faces are specified as two half faces. For example, w/s, is a face with a weak top and a strong bottom. Leaving out the slash indicates that the data were averaged regardless of top or bottom status. For example, ws specifies all faces with one weak and one strong half, regardless of location (i.e., w/s and s/w). This notation averages over family. Where appropriate, these factors will be designated with subscripts. For example, w_Jones_/s_Jones_ is a face with a weak Jones top and a strong Jones bottom.

#### Target Faces

**Fig 1:**
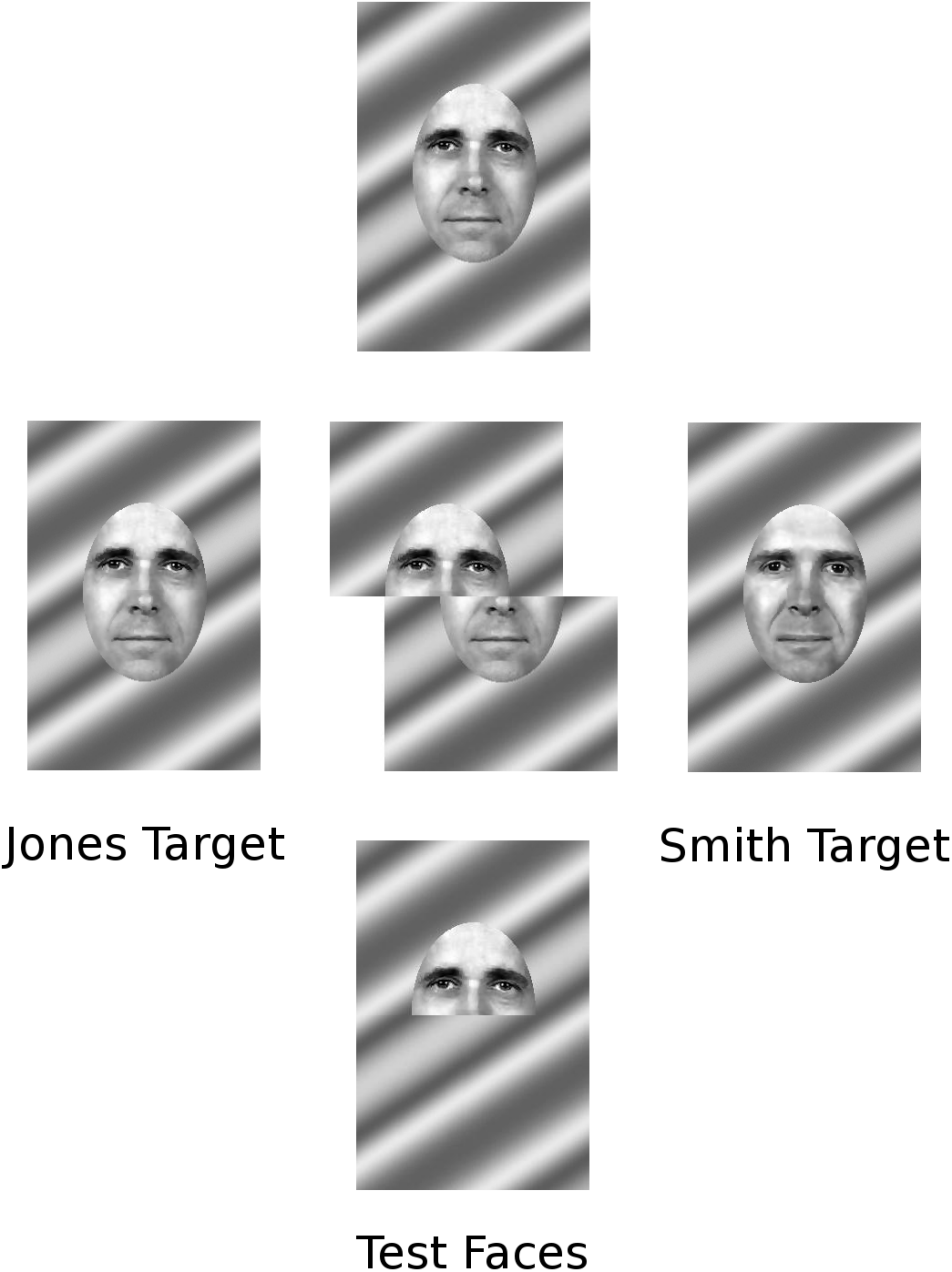
Target faces and sample together whole, split whole, and half test faces.

All of the stimuli used in the experiment were derived from two gray-scale target faces, the Jones and Smith targets. Two faces were selected from the FERET database [49]. These faces were warped so that their major facial features aligned and were then cropped to remove the hair and head outline. Using pilot data, the bottom halves of the two faces were morphed so that the discriminability of the top and bottom half faces were roughly equivalent. The cropped areas of the 256 × 384 pixel images were filled with a sinusoidal grating. See Fig 1 for the targets.

#### Stimulus Calibration

The purpose of calibration was to determine the morph levels that provide weak, medium, and strong evidence for the Jones and Smith targets individually for each participant. A staircase algorithm was used to find a medium morph level for each half face and for each family. Linear interpolation from this medium level was used to determine weak and strong levels.

The top and bottom halves of each target face were calibrated separately. Consider calibration for the top half of the upright Jones target. On each trial, participants were shown a top half face flanked by the two targets and were asked to select the target face it more closely resembled. Feedback was given after each response. Because this is calibration for the top half of the Jones face, a response of Jones was considered correct. A 2-up, 1-down staircase algorithm was used to find the morph level that produced roughly 71% accuracy [50]. In particular, the proportion of Jones present in the morph was reduced by 5.6% (minimum of 50%) after every two consecutive correct responses and was increased by 5.6% (maximum of 100%) after each incorrect response. This process continued for 18 trials. The half face morph on the first calibration trial was very similar to the Jones target face (a 94% Jones, 6% Smith morph). Calibration of the other half faces proceeded independently, in a similar fashion.

The staircases for the top and bottom halves for Jones and Smith were randomly intermixed. There were 72 (2 top/bottom × 2 Jones/Smith × 18 repetitions) half-face trials. To reduce the probability that participants would develop strategies tailored only to half faces, 48 whole-face trials (24 together and 24 split faces of fixed morph levels) were randomly interspersed with the half-face trials. The whole-face trials did not affect the half-face staircases or any further analysis.

This procedure resulted in top and bottom half morph levels for each target that produced an intermediate level of accuracy. These morph levels were used as the medium strength. On the basis of pilot data, weak levels were defined as the morph midway between the medium level and a 50% morph and strong levels were defined as the morph two-thirds between the medium level and a 100% morph. For example, if the medium level for the top face Jones target was an 82% Jones morph. Then the weak level would be a 66% Jones morph and the strong level would be a 94% Jones morph.

#### Test Faces

The 56 test faces were generated from the calibrated weak, medium, and strong levels as follows. The test faces are subdivided into half, whole, and opposite faces each of which can be together or split.

Twelve (3 morph levels × 2 top/bottom × 2 Jones/Smith) *half* test faces were generated directly from the weak, medium, and strong calibration morph levels. Eighteen (3 top morph levels × 3 bottom morph levels × 2 Jones/Smith) *whole* test faces were created by crossing the w/, m/, and s/ top-half faces for each target with the /w, /m, and /s bottom-half faces for that same target, resulting in whole faces in which the two halves support the same response. Four (2 top morph levels × 2 top Jones/Smith) *opposite* faces combined m and w evidence from opposite families, and were included as a check that the half faces from the weaker category were not taken as evidence for the stronger category. In the figures, opposite faces are designated as wom–weak, opposite medium. As was done for the targets, all test faces were superimposed on a sinusoidal grating.

The 22 whole and opposite test faces were then formatted to encourage automatic or controlled processing. In the *together* configuration, top and bottom halves aligned normally. In the s*plit* configuration, there was a sixty pixel offset between top and bottom halves. To emphasize that the two halves were originally from the same face, the background was split along with the face, giving the impression of a single edited image. Because there was no effect after extensive pilot testing, half faces were not split. Sample test faces are provided in Fig 1.

### Procedure

The experiment was broken into two phases, the calibration phase and the integration phase.

The calibration phase was described previously. During each trial of the integration phase, participants viewed a test face flanked by the two target faces. After a two-second presentation, the face was masked by a scrambled sets of target-face features. After an additional 250ms, participants were first asked to select the target that better resembled the test face and then to provide “the likelihood that you are correct” on a 6-point scale from 50% to 100%. No feedback was provided on the integration trials. For whole faces, participants were told to treat both halves of an image as coming from a single face, even in the split condition.

During each integration block, each test face appeared once, with the exceptions of w/w, m/m, and s/s stimuli, which appeared twice, for a total of 68 trials per block (12 half, 36 whole, 8 opposite, and 12 additional w/w, m/m, s/s faces). Participants completed two sessions of the experiment on separate days. During the first session they completed calibration and one integration block. During the second session, they completed three integration blocks. In separate experimental blocks, the participants were also calibrated and tested on upside-down versions of the stimuli. Data from these trials are not discussed.

To randomize starting eye fixations relative to the stimuli and thereby reduce a potential top/bottom starting bias, trials began with a test face appearing in one of nine positions on the screen. These positions were defined by crossing three vertical locations (0, +100, -100 pixel offset from y-center) with three horizontal locations (0, +100, -100 pixel offset from x-center).

## Results

The present analysis focuses on participants’ choice proportions during the integration phase. Confidence judgments produced a very similar pattern of results and are provided in the Appendix.

Trials with a confidence rating of 50% or a response time of less than 150ms or greater than 5s were removed from analysis. This procedure removed approximately 13% of all trials. 2.25% of together trials and 2.91% of split trials had a confidence rating of 50%.

In the whole and half face conditions, responses were coded as correct if they matched the appropriate target face. For example, given a w_Jones_/s_Jones_ test face, Jones was the correct response. For opposite faces, where the top and bottom halves favor different responses, the correct response was the one supported by the stronger evidence. For example, given a m_Jones_/w_Smith_ test face, Jones was the correct response.

**Fig 2:**
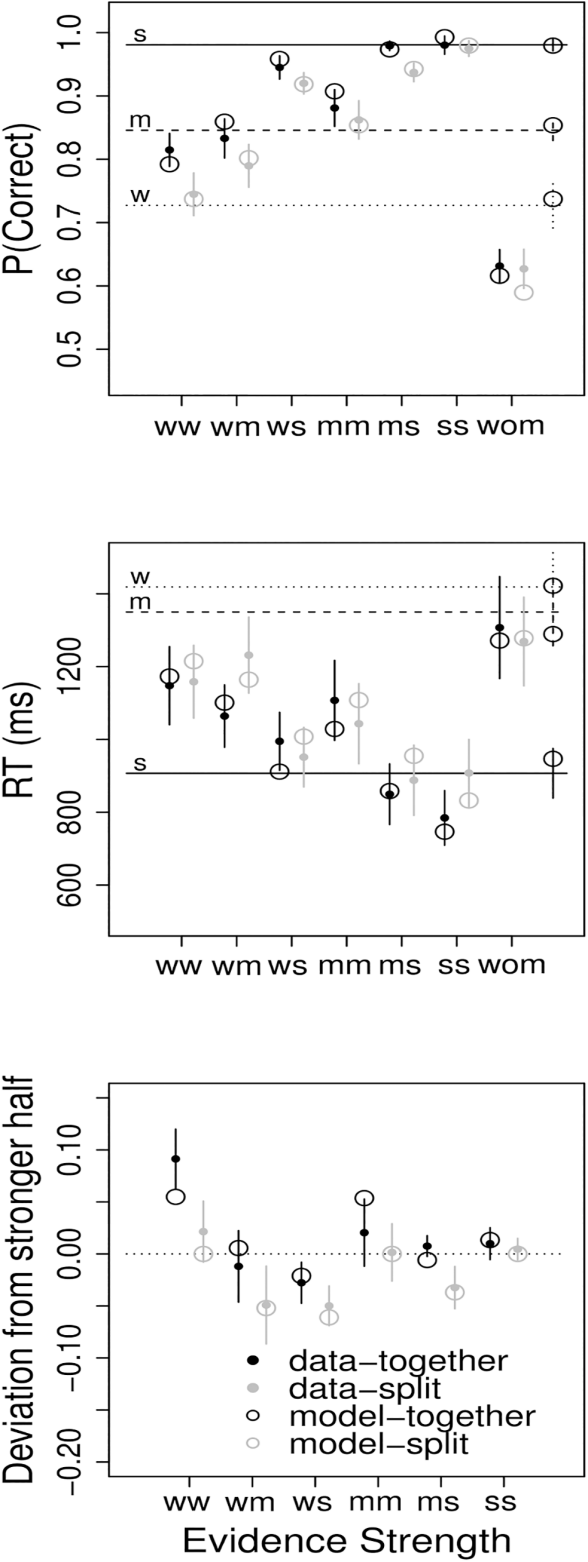
Data and model predictions for accuracy (top), response time (middle), and deviation scores (bottom) for weak (w), medium (m), and strong (s) half faces and averaged within the weak-weak (ww), weak-medium (wm), weak-strong (ws), medium-medium (mm), medium-strong (ms), strong-strong (ss), and weak-opposite medium (wom) together and split whole-face conditions. Error bars are between-subject standard errors. The far right error bars and circles in the top two panels are the standard errors and model predictions for the half faces.

The main graphs of Fig 2 shows the accuracy, response time, and deviation scores for each combination of evidence strength separately for the together and split conditions. For display purposes, the data were collapsed across family and top and bottom half faces.

Whole face accuracy, response times, and dilution were initially analyzed using linear mixed-effects models ([51, 52]). The following fixed effects were included in the model: together/split (coded as-.5 and .5), top and bottom face strength (weak = -1, medium = 0, strong = 1), and all interactions. Because they were not of theoretical interest, block, trial, and family were not included in the model. Including these factors as fixed, main effects, however, had no qualitative effect on the reported results. Subjects were included as a random effect with a random intercept. For accuracy, a logistic model was used to model performance on each trial. Model comparison with the full model was used to determine *p*-values. The results of these analyses are provided in Table 1 and will be discussed below.

**Table 1:** Results of the linear mixed-effects models for accuracy, response time, and deviation score. *Note*. The *χ* ^2^ and *p* values were determined by comparison with the full model. All *χ* ^2^ df = 1. Bold = p < .05.

	Estimate	S.E.	*t-*, *z-*value*	*χ* ^*2*^	*p*
Accuracy					
**(intercept)**	**2.49**	**0.18**	**14.07**	**43.48**	**< .001**
**together/split**	**-0.45**	**0.14**	**-3.21**	**10.74**	**.001**
**top strength**	**0.72**	**0.09**	**8.43**	**78.98**	**< .001**
**bottom strength**	**0.71**	**0.08**	**8.35**	**77.24**	**< .001**
together/split×top strength	-0.10	0.17	-0.57	0.33	.57
together/split×bottom strength	-0.14	0.17	-0.81	0.67	.41
top strength×bottom strength	0.14	0.10	1.35	1.84	.17
together/split×top strength×bottom strength	-0.10	0.20	-0.50	0.25	.62
Response time					
**(intercept)**	**1.01**	**0.08**	**12.31**	**42.63**	**< .001**
together/split	0.03	0.03	1.07	1.15	.28
**top strength**	**-0.11**	**0.02**	**-6.04**	**36.32**	**< .001**
**bottom strength**	**-0.09**	**0.02**	**-5.17**	**26.64**	**< .001**
together/split×top strength	0.01	0.03	0.17	0.03	.86
together/split×bottom strength	0.00	0.03	-0.08	0.01	.94
top strength×bottom strength	0.00	0.02	0.04	0.00	.97
together/split×top strength×bottom strength	0.04	0.04	0.90	0.81	.37
Deviation score					
(intercept)	-0.01	0.01	-0.49	0.25	.61
**together/split**	**-0.03**	**0.01**	**-2.85**	**8.11**	**.004**
top strength	-0.01	0.01	-1.69	2.86	.09
bottom strength	-0.01	0.01	-1.00	1.01	.32
together/split×top strength	0.01	0.01	0.95	0.90	.34
together/split×bottom strength	0.01	0.01	0.52	0.26	.61
**top strength×bottom strength**	**0.04**	**0.01**	**4.54**	**20.57**	**< .001**
together/split×top strength×bottom strength	-0.01	0.02	-0.31	0.09	.76

*z-scores for accuracy, *t*-scores for response time and deviation score.

### Accuracy

Quick checks of the accuracy data (Fig 2, top panel) suggest that the strength levels were appropriately calibrated. Half-face accuracy was higher than desired, but was ordered correctly: w < m < s. Both together and split ww, mm, and ss whole face accuracy increased in that order. The mean accuracies for both together and split wom trials were greater than chance, *t*(18) = 5.07, *p* < .001, *d* = 1.16 and *t*(18) = 4.11, *p* < .001, *d* = 0.94, and less than the mean accuracy for medium half faces, *t*(18) = 8.27, *p* < .001, *d* = -1.90 and *t*(18) = 7.07, *p* < .001, *d* = -1.62, confirming that the weak half faces were taken as evidence for the appropriate category. As expected, the opposite faces yielded the lowest accuracy, suggesting that participants perceived top and bottom halves as supporting opposite responses. These trials were included only as a manipulation check and will not be discussed further. Accuracy increased as the strength of the top- and bottom-faces increased. Accuracy on split faces was significantly lower than accuracy on together faces (.87 vs. .91). A more direct test of differences in dilution across conditions is provided below.

### Response Times

Although there were no strong predictions for the effect of format on response times (Fig 2, middle panel), split faces are both unfamiliar and more likely to invoke controlled processing potentially slowing processing and increasing response times. Although the trend is slightly in that direction (for whole faces, mean *RT*_*together*_ = 993ms, mean *RT*_*split*_ = 1025ms), the effect of split on response time was not significant. Response times, however, did decrease as the strength of the top- and bottom-faces increased. The response times are presented primarily as an additional constraint on the model, as described below.

### Deviation Scores

The primary research question concerns the conditions under dilution would occur. To address this question, whole face accuracy was compared to the accuracy of the stronger half face alone. The stronger half faces were determined using each individual's mean half-face performance, which agreed with the experimenter labels for 87% of faces. Deviation scores for each whole test face for each participant were calculated by subtracting the mean accuracy for the stronger half face—i.e., the half face present in the whole face that produced the greatest accuracy when alone—from the accuracy of each whole face response. For example, consider a correct response to the presentation of a w/s Jones face. Further, assume that the mean accuracy for the w/ and /s half faces were 0.6 and 0.8, respectively. Then the deviation score for this response would be 1–0.8 = 0.2. A deviation score less than 0 indicates a dilution. When the deviation score is greater than 0, weak evidence is adding to strong evidence.

Together deviation scores were significantly higher than split deviation scores (0.014 vs. -0.019). Although deviation scores did not significantly change with top- and bottom-half strength, these factors did interact. In the bottom panel of Fig 2, it is clear that deviation scores decreased as top and bottom strengths diverged. For example, the effect of a strong half face depends on the strength of the other half—the deviation score for a ws face is much lower than the deviation score for an ss face. To explore this interaction further, the mean deviation score for each subject and each difference in top and bottom strength (i.e., ww = mm = ss = -1, wm = ms = 0, ws = 1) was calculated. A linear mixed-effects model was run with together/split, strength difference, and the interaction as fixed effects and subjects as a random factor with a random intercept. As previously, there was a main effect of together/split (est. = -0.03, s.e. = 0.01, *t*-value = -2.39, *χ* ^2^(1) = 5.71, *p* = .02), with split faces producing greater dilution. Critically, deviation scores decreased as the strength difference increased (est. = -0.03, s.e. = 0.01, *t*-value = -3.96, *χ* ^2^(1) = 14.93, *p* < .001), suggesting that dilution increases with the difference in evidence strength across sources. No other effects were significant.

The previous analysis suggests that evidence is combined less efficiently for split faces. The data, however, also suggest that a stronger form of dilution may occur under certain conditions. That is, weak evidence may actually reduce the effect of strong evidence. Note that the split ws faces most closely match the standard dilution effect stimuli, i.e., strong evidence paired with weak evidence. A post-hoc contrast found that the deviation score of the split ws faces was significantly below zero, *t*(18) = 2.61, *p* = .02, *d* = -0.60, suggesting that the weak evidence reduced the effect of the strong evidence. Conversely, optimal integration of information would create positive deviation scores. We expected together faces to be more likely to show additive information integration, however, the closer performance is to ceiling, the more difficult it is to improve. For these reasons, we predicted that the together ww condition should produce reliable additive effects. Indeed, these faces yielded significant positive deviation scores, *t*(18) = 3.19, *p* = .005, *d* = 0.73.

Another method for investigating the presence of automatic vs. controlled processing is to look at the relationship between response times and deviation scores. We used each individual’s median response time to classify each whole face trial as either fast or slow. Across all whole face trials, deviations scores were significantly lower for slow trials, *t*(18) = 5.47, *p* < .001, *d* = 0.07. This effect held for together trials, *t*(18) = 6.35, *p* < .001, *d* = 0.07, as well as split trials, *t*(18) = 3.71, *p* < .01, *d* = 0.06.

### The Multi-Component Information Accumulation Model

The Multi-Component Information Accumulation model (McIA) was developed based on the assumption that information integration is governed by a process of accumulating evidence from multiple sources until a decision threshold is reached. The McIA is implemented as a random walk. Evidence accumulates towards two response thresholds–one for the correct response and one for the incorrect response. On every time step, a source of evidence is sampled and the random walk probabilistically takes a step towards the threshold supported by the evidence. There are three sources of evidence: the top half face alone, the bottom half face alone, and the whole face. The model typically extracts information separately from the top and bottom halves. The perceptual system, however, occasionally short-circuits this process by automatically, and optimally, integrating the evidence from the two halves. The whole face evidence source is intended to represent this automatic information integration. The accumulation process continues until a response threshold is reached, at which point the associated choice is made. The predicted response time is the number of steps taken to reach the threshold.

### Model Implementation

On half-face trials, there is only one possible source of evidence, the half face. On whole face trials, attention switches back and forth between the three sources of information. At each moment in time, evidence is sampled based on an attention parameter, *α*, which is the probability of automatically integrating information from the two halves. Under the assumption that whole-face information integration is disrupted by the splitting the faces, *α* is set to 0 for split faces (allowing a non-zero *α* for the split faces did not significantly improve the quantitative fits, wSSE = 17.77, and *α* was small, *α* = .069). The *α* parameter is the only difference between together and split model predictions. The probabilities of sampling from the top half or the bottom half, (1 - *α*)/2, are assumed to be equal.

Evidence strength determines the probability that the process will take a step toward the correct decision threshold. The probabilities that the random walk will take a step towards the correct threshold given weak, medium, and strong half-face evidence are *δ*_*weak*_, *δ*_*medium*_, and *δ*_*strong*_, respectively. The probability of moving towards the correct threshold given whole face evidence is calculated as the optimal combination of the top and bottom probabilities. In particular, the optimal combination is given by:

$$\delta_{whole}=\frac{\frac{\delta_{top}}{\left(1-\delta_{top}\right)}\times \frac{\delta_{bottom}}{\left(1-\delta_{bottom}\right)}}{1+\frac{\delta_{top}}{\left(1-\delta_{top}\right)}\times \frac{\delta_{bottom}}{\left(1-\delta_{bottom}\right)}}$$ (1)

This equation, based on the optimal integration of information from Massaro [17], reflects the idea that whole face evidence is the result of the automatic perceptual integration of visual information and is extremely effective and accurate. For example, consider a w/s whole face with *δ*_*weak*_ = .55 and *δ*_*strong*_ = .75. Then

$$\delta_{whole}=\frac{\frac{.55}{\left(1-.55\right)}\times \frac{.75}{\left(1-.75\right)}}{1+\frac{.55}{\left(1-.55\right)}\times \frac{.75}{\left(1-.75\right)}}=.81,$$

which is more likely to move the random walk toward the correct boundary than either half face alone. In contrast, switching between top and bottom half faces effectively averages the two probabilities (.55 + .75)/2 = .65, yielding a value lower than the probability associated with the stronger half face (i.e., .75). Thus, use of the whole face information can produce the additive information integration seen in perceptual studies and relying on half face information alone can produce dilution.

The threshold parameter, *θ*, is assumed to be equal for each family, thus the initial distance to the correct and incorrect boundary is the same. Random walk models measure time in steps. A linear transformation is used to translate steps to milliseconds. That is, *RT* = *τ* + *kN*, where *τ* is a constant, non-decision time parameter, *k* is the time taken to make each step, and *N* is the number of steps to threshold. It turns out that the response times for half faces were considerably longer than response times for whole faces with comparable accuracy. One possible reason for this difference is that comparing a half face to a whole face target is an additional processing disruption. To accommodate this possibility, a half-face multiplier, *h*, further scaled the time it took to make each step in the random walk. Although we are largely agnostic regarding this issue, *h*, can be interpreted as representing the time required to manipulate the target faces, which are whole, into a format that can be easily compared to a half test face. That is, the response time for a half face is given by *RT* = *τ* + *hkN*. Other possible models were tried including increasing the thresholds, wSSE = 25.57, and decreasing the rates, wSSE = 48.09, for half faces. None of these models fit as well as including a multiplier on *k*. As this half-face effect was unexpected and not the main focus of this article, we leave exploration of this effect to future research.

Conveniently, the McIA model has closed-form analytical solutions for choice probabilities and response times (see [53, 54]), allowing us quickly calculate model predictions without the need for simulations. The McIA has the eight free parameters listed in Table 2. These eight parameter were used to account for the 34 data points from the top and middle panels of Fig 2 (12 whole face, 2 opposite face, and 3 half face accuracies and response times). The model was fit to the data using a weighted sum of squares error (wSSE) fit measure. The error was based on accuracy, response time, and, because they are of particular interest, deviation scores. Although the deviation scores are redundant with accuracy they were included to ensure that the model accounted for these relatively small, but theoretically important effects. To put accuracy, response time, and deviation scores on comparable scales, the error scores were standardized. In particular, the difference between each data point and the associated model prediction was divided by the standard error of the mean for that data point. To create the overall fit measure, these differences were squared and then summed. The best fitting parameters were found using the Nelder-Mead simplex [55] algorithm in Octave [56].

**Table 2:** Best Fitting Parameters for the McIA Model.

Parameter	Value	Definition
*θ*	6.76	Response threshold.
	.30	Probability of using optimal information integration for together faces.
*δ*_*weak*_	.54	Probability of moving toward the correct response threshold given weak, medium, or strong evidence.
*δ*_*medium*_	.56	
*δ*_*strong*_	.64	
	359	Non-decision time (ms).
*k*	20	Step time (ms).
*h*	1.24	Half-face multiplier.

### Modeling Results

The best fitting (wSSE = 18.61) accuracy, response time, and deviation score model predictions are shown as open circles in Fig 2. The best fitting parameters are given in Table 2.

The model does an excellent job of accounting for the data. First, consider the accuracy data (top panel of Fig 2). The McIA accounts for all of the qualitative differences across evidence strength. By using the more accurate whole-face information in the together conditions, the model can also predict the relative together condition advantage.

Second, the model captures all of the major response time trends (middle panel of Fig 2). The model, however, does predict a slight response time advantage for the together faces. This prediction follows directly from the accuracy advantage. Because the whole-face information strongly favors the correct response, the random walk is more likely to move towards the correct threshold both increasing accuracy and reducing the number of steps taken to reach the threshold. Further studies will be needed to determine the validity of this prediction.

Finally, and most important, the model captures the basic qualitative patterns in the deviation scores (bottom panel of Fig 2). Given the relatively subtle nature of these differences, this result is especially impressive. Due to a lack of whole-face evidence in the split condition, the model correctly predicts that split deviations scores will be lower (more negative) than together deviation scores. Related to this prediction, reliance on whole-face information allows for additive information-integration in the together conditions. In particular, the model can account for the relatively high deviation score for the ww faces. The model also correctly predicts that deviation scores will decrease with an increase in strength difference between the two sources of evidence. For example, within the together and split conditions, the lowest deviation scores are for the ws stimuli and the highest deviation scores are for the ww stimuli. This prediction stems from the half-face sampling process. Because both halves are sampled with equal probability, weaker evidence will greatly slow progress towards the correct threshold, relative to stronger evidence alone, resulting in much lower accuracy. A particularly interesting prediction from the model, mirrored in the data, is that trials with two similar sources of information (i.e., ww, mm, and ss) cannot have deviation scores substantially below zero. In these conditions, the probability of moving towards the correct threshold using both sources of evidence is approximately as high as the probability for the half faces in isolation. The deviations scores for these conditions are not significantly below 0 (*p* > .1).

The McIA framework can also be used to compare models that optimally combine evidence with models that only average evidence. An assumption of optimal integration was implemented by assuming *α* = 1 for all conditions. That is, evidence from both halves of a whole face was always combined according to Eq 1. A lack of whole-face integration was implemented by assuming *α* = 0 for all conditions. The results are provided in the S1 File. Here we highlight the major findings. Both models miss certain qualitative aspects of the data. First, when *α* cannot vary across conditions, the model is unable to differentiate split and together performance. Second, the *α* = 1 model tends to over-predict both accuracy and deviation scores, resulting in a relatively poor quantitative fit. Finally, and most important, neither model can account for the deviation scores. When *α* = 1, all deviation scores are restricted to be non-negative, so this model cannot produce the dilution seen in stimulus such as split ws. When *α* = 0, all deviation scores must be non-positive, so this model cannot predict the additive performance for conditions such as together ww. Inclusion of the *α* parameter is the key to accounting for the full range of qualitative data patterns. For completeness, a model with *α*_*split*_ = 0 and *α*_*together*_ = 0 was also fit and is provided in the S1 File. This model does a poor job quantitatively predicting the data.

Another possibility is that the same integration processes are at work in both the together and split conditions, but that the unusual split format causes a general reduction in the information accumulation rate. To implement this idea, for the split faces, each *δ* was multiplied by a parameter between 0 and 1. Within this framework, information can be averaged or can be combined optimally. If the two sources of evidence are averaged (i.e., *α* = 0 for all conditions), the model cannot account for the additive integration seen in condition ww. Therefore, we started from an assumption of optimal integration (i.e., *α* = 1 for all conditions). The results are shown in Fig B and Table A of the S1 File. Although this model can account for the difference between together and split faces, it fails to capture the quantitative pattern of accuracies and deviation scores. In particular, it greatly overestimates accuracy in the together condition.

## Conclusions

The overarching goal of this research was to determine the processes involved in combining multiple sources of information. This research represents a synthesis of two divergent trends in the literature: perception, in which information integration is often assumed to be (near) optimal, and judgment and decision making, in which integration is often assumed to rely on heuristics that produce systematic violations of optimality. The present experiment is a first step at reconciling these contrasting results within a common experimental paradigm. The basic idea is that information integration may be modulated by stimulus presentation. In particular, the current research tests the hypothesis that perceptual information will be more resistant to the dilution effect than information that is combined in a more cognitive fashion.

Observers categorized perceptual stimuli, faces, in which each half-face provided different levels of evidence for the correct response. Automatic processing was encouraged by presenting faces in a normal configuration. Splitting the faces horizontally was meant to inhibit this automatic process and encourage participants to form separate judgments of the two half faces, which would then be combined in a more controlled manner. In line with these predictions, participants were more accurate when faces were presented together. More importantly, there was strong qualitative evidence of additivity in some together conditions, in which weak information adds evidence, and dilution in some split conditions, in which weak information reduces evidence relative to a stronger source.

The results of this experiment suggest that cases of dramatically sub-optimal information integration are not limited to those involving the numerical or linguistic stimuli found in traditional judgment and decision making research. Although sub-optimality in perception is well documented, the bulk of the extant literature on perceptual information integration suggests that two sources of evidence in favor of the same response should produce more accurate responses than either source alone. Instead, there were conditions under which observers were less accurate when given additional diagnostic evidence. This result does not rule out models that include a near-optimal component, but rather suggests that any such model would also require some process that naturally produces negative deviation scores.

Indeed, the overall quantitative and qualitative data patterns were well predicted by the Multi-component Information Accumulation model, which assumes that evidence accumulates from either each half face alone or, for together faces, the optimal combination of the two half faces. Reliance on only the half faces produces averaging behavior and dilution, while optimal integration can produce additive results. Because both positive and negative deviation scores were observed, neither process in isolation can account for the data; both processes are necessary to account for the full pattern of results. The success of the McIA suggests that the distinction between automatic and controlled processing might go some way towards reconciling the different patterns of data observed in the decision making and perception literatures.

We recognize that these results only provide indirect support for the role of automatic and controlled processing in information integration. Although the experiment was designed—drawing from extensive integration research in perception, cognition, and judgment—to selectively encourage automatic or controlled processing, it may be possible to otherwise explain the findings. Additionally, it is likely that both processes were active throughout the task, though in different degrees, depending on the condition. In this sense, processing mode can be thought of as a continuum, with different stimuli, task demands, cognitive factors, etc. determining the degree to which an individual will use more automatic or more controlled integration strategies. Despite these limitations, the results of our experiment and modeling clearly indicate progress toward reconciling the divergent findings in the information integration literature. Regardless of the underlying explanation, our manipulations were able to produce both additive effects—reminiscent of the near-optimal integration commonly assumed in models of perception—and dilution effects—as frequently seen in traditional judgment tasks—within a common paradigm and so provide valuable insight into the factors that can lead to dramatic and systematic sub-optimal performance in information integration. On this foundation, future research into the factors controlling the quality of information integration can be built.

## Supporting Information

S1 File Fig A in S1 File. Confidence data for weak (w), medium (m), and strong (s) half faces and averaged within the weak-weak (ww), weak-medium (wm), weak-strong (ws), medium-medium (mm), medium-strong (ms), strong-strong (ss), and weak-opposite medium (wom) together and split whole-face conditions. Error bars are between-subject standard errors. The far right error bars in the top two panels are the standard errors for the half faces. Fig B in S1 File. Data and alternate model predictions (see text for details) for accuracy (top), response time (middle), and deviation scores (bottom) for weak (w), medium (m), and strong (s) half faces and averaged within the weak-weak (ww), weak-medium (wm), weak-strong (ws), medium-medium (mm), medium-strong (ms), strong-strong (ss), and weak-opposite medium (wom) together and split whole-face conditions. Error bars are between-subject standard errors. The far right error bars and circles in the top two panels are the standard errors and model predictions for the half faces. Table A in S1 File. Fit Values and Best Fitting Parameters for the alternative models in Fig B in S1 File. (DOCX)
