# Supplementary material for: The Dilution Effect and Information Integration in Perceptual Decision Making
Source: PLoS One. 2015 Sep 25;10(9):e0138481. doi: 10.1371/journal.pone.0138481 (PMC4583276; doi:10.1371/journal.pone.0138481)
Supplement: S1 File — (DOCX) [file pone.0138481.s001.docx]

**Supporting Information**





S1 Fig A. Confidence data for weak (w), medium (m), and strong (s) half faces and averaged within the weak-weak (ww), weak-medium (wm), weak-strong (ws), medium-medium (mm), medium-strong (ms), strong-strong (ss), and weak-opposite medium (wom) together and split whole-face conditions. Error bars are between-subject standard errors. The far right error bars in the top two panels are the standard errors for the half faces.





S1 Fig B. Data and alternate model predictions (see text for details) for accuracy (top), response time (middle), and deviation scores (bottom) for weak (w), medium (m), and strong (s) half faces and averaged within the weak-weak (ww), weak-medium (wm), weak-strong (ws), medium-medium (mm), medium-strong (ms), strong-strong (ss), and weak-opposite medium (wom) together and split whole-face conditions. Error bars are between-subject standard errors. The far right error bars and circles in the top two panels are the standard errors and model predictions for the half faces.

S1 Table. Fit Values and Best Fitting Parameters for the alternative models in S1 Fig B.

|  | **Model** | | | |
| --- | --- | --- | --- | --- |
|  | **α_s_ = 0, α_t_ = 0** | **α_s_ = 0, α_t_ = 1** | **α_s_ = 1, α_t_ = 1** | **δ reduction** |
| wSSE | 56.139 | 80.684 | 194.621 | 86.885 |
| θ | 19.611 | 29.646 | 2.341 | 1.4811 |
| δ_weak_ | 0.51452 | 0.50696 | 0.56356 | 0.64239 |
| δ_medium_ | 0.52486 | 0.51324 | 0.63724 | 0.73415 |
| δ_strong_ | 0.55931 | 0.53451 | 0.84885 | 0.93229 |
| τ | 469 | 621 | 433 | .006 |
| k | 2 | 0.7 | 157 | 566 |
| h | 1.37 | 1.2973 | 1.1058 | 1.1042 |
| δ multiplier | - | - | - | 0.87949 |
